# Risk-Adjusted Survival after Coronary Artery Bypass Grafting: Implications for Quality Improvement

**DOI:** 10.3390/ijerph110707470

**Published:** 2014-07-21

**Authors:** Jimmy T. Efird, Wesley T. O’Neal, Stephen W. Davies, Jason B. O’Neal, Linda C. Kindell, Curtis A. Anderson, W. Randolph Chitwood, T. Bruce Ferguson, Alan P. Kypson

**Affiliations:** 1 Department of Cardiovascular Sciences, Brody School of Medicine, East Carolina Heart Institute, East Carolina University, Greenville, NC 27834, USA; E-Mails: jimmy.efird@stanfordalumni.org (J.T.E.); kindelll@ecu.edu (L.C.K.); andersoncu@ecu.edu (C.A.A.); chitwoodw@ecu.edu (W.R.C.); fergusont@ecu.edu (T.B.F.); kypsona@ecu.edu (A.P.K.); 2 Statistical Analysis Unit, Center for Health Disparities, Brody School of Medicine, East Carolina University, Greenville, NC 27834, USA; 3 Department of Internal Medicine, Wake Forest School of Medicine, Winston-Salem, NC 27157, USA; E-Mail: woneal@wakehealth.edu; 4 Department of General Surgery, University of Virginia School of Medicine, Charlottesville, VA 22908, USA; 5 Department of Anesthesia, Critical Care, and Pain Medicine, Beth Israel Deaconess Medical Center, Harvard Medical School, Boston, MA 02215, USA; E-Mail: jboneal@bidmc.harvard.edu

**Keywords:** outcomes, coronary artery bypass grafting, CABG, survival, mortality

## Abstract

Mortality represents an important outcome measure following coronary artery bypass grafting. Shorter survival times may reflect poor surgical quality and an increased number of costly postoperative complications. Quality control efforts aimed at increasing survival times may be misleading if not properly adjusted for case-mix severity. This paper demonstrates how to construct and cross-validate efficiency-outcome plots for a specified time (e.g., 6-month and 1-year survival) after coronary artery bypass grafting, accounting for baseline cardiovascular risk factors. The application of this approach to regional centers allows for the localization of risk stratification rather than applying overly broad and non-specific models to their patient populations.

## 1. Introduction

Current models to predict outcomes after coronary artery bypass grafting (CABG) have been developed using statewide and national data [1,2]. However, predicted survival estimates from these databases tend to focus on hospitals in large urban areas, missing many rural regions with racially and economically diverse populations. Furthermore, prediction models are more likely to perform poorly when applied to groups or regions other than those in which they were derived.

Models customized for regional centers or specific areas with unique patient populations are known to perform better than generalized models developed using patient data that is unrepresentative of the targeted population [3]. Accordingly, localized models are important for physicians to optimize individual postoperative care and to appropriately inform patients of their likelihood of survival after surgery.

The development and application of individual institution quality measurements allow for constant evaluation and outcome improvement. To aid surgeons and centers, we have developed a simple graphical technique to examine risk-adjusted survival estimates that account for case-mix severity. This technique, displayed as efficiency-outcome plots, enables regional centers to examine their outcomes over contiguous time periods. It also fills an important gap in the quality assessment literature (e.g., graphical tools for monitoring surgical performance) by taking into account censored, time-to-event data. In this paper, we present the application of this graphical procedure with data from a large tertiary referral heart institute.

## 2. Experimental Section

The Institutional Review Board at the Brody School of Medicine, East Carolina University, approved the analysis. Details of the database have been previously described and are summarized below [4,5,6,7,8,9,10,11,12].

### 2.1. Patients and Variables

The data used in this example analysis included patients undergoing first-time, isolated CABG at the East Carolina Heart Institute between 1 January 2001 and 31 December 2008. Patients were categorized into 2-year increments by date of surgery. Demographic data, comorbid conditions, coronary artery disease (CAD) severity, and surgical data were collected at the time of surgery. The analysis was restricted to black and white patients to minimize the potential for residual confounding (~1% other races). Racial identity was self-reported. Emergent cases were considered to have a different etiology following surgery and were excluded in our example (*n* = 97).

### 2.2. Definitions

Mortality was defined as any cause of death postoperatively. CAD was defined as ≥50% stenosis and confirmed by angiography before surgery. Postoperative complications including operative mortality were defined as occurring within 30 days following CABG in or out of our hospital and after 30 days during the same hospitalization following surgery.

### 2.3. Setting

The East Carolina Heart Institute is a tertiary care, population-based heart hospital located in eastern North Carolina, a rural region with a large economically impoverished population [4,5,6,7,8,9,10,11,12]. The institute is the largest stand-alone facility devoted to cardiovascular care in the state of North Carolina. Cardiovascular disease is the leading cause of death in North Carolina with an unequal burden occurring in eastern North Carolina [13]. The majority of patients treated at the East Carolina Heart Institute live and remain within a 150-mile radius of the medical center.

### 2.4. Data Collection and Follow-up

The main sources of data were the Society of Thoracic Surgeons (STS) Adult Cardiac Surgery Database linked with the electronic medical record at the Brody School of Medicine. The National Death Index was used to obtain death dates for patients lost to follow-up and also used to validate death information collected in our electronic medical record. Linkage with the National Death Index was performed using a multiple criteria, deterministic matching algorithm [14]. Less than 5% of validated deaths failed to correctly match with the National Death Index. The Epidemiology and Outcomes Research Unit at the East Carolina Heart Institute regularly perform data quality and cross-field validation.

### 2.5. Statistical Analysis

Categorical variables were reported as frequency and percentage; continuous variables were reported as mean (plus or minus 1 standard deviation), median, and range. Trend across time periods was assessed using the Cochran-Armitage test for binary variables and a standard linear regression test (H_o_: β_1_ = 0) for continuous variables. For categorical variables with more than two stratification levels, a chi-square test for non-zero correlation was used to assess trend.

Risk-adjusted survival estimates at 6 months and 1 year were computed using the multivariable product-limit method with date of surgery as the reference point [15]. Patients who were still alive at the date of last contact were censored. The family of product-limit models is known to have strong uniform consistency and other desirable statistical properties [16,17,18]. When there are no covariates, the multivariable product-limit model approximates the actuarial survival curve [15,18,19]. For cross-validation (model performance), we computed the percentage difference of fitted probabilities for each time period under the multivariable product-limit model to observe probabilities measured using standard survival curves. Percentage difference values within ±2.5% were considered to be in the equivalence zone. A Wilcoxon-signed rank sum test was used to test the null hypothesis that the general measure of central tendency for the percentage differences did not differ from zero. The discriminate abilities of our models were assessed by examining estimated survival plots of key variables for risk group separation and also by computing the c-statistic concordance probability given censoring [20,21,22]. An interaction of model variables by time may affect overall fit and performance of the model. A test for this assumption was performed by including time-dependent covariates in our regression model [23]. The models included variables that have been previously reported to be associated with cardiovascular-related mortality, regardless of their statistical significance in our dataset [4,5,6,7,8,9,10,11,12]. These included age, sex, race, hypertension, CAD severity, heart failure, and prior stroke. Efficiency-outcome plots were generated to illustrate risk-adjusted survival by time period. Statistical significance was defined as *p* ≤ 0.05. SAS Version 9.3 (SAS Institute: Cary, NC, USA) was used for all analyses.

## 3. Results and Discussion

### 3.1. Results

A total of 4639 patients underwent CABG between 2001 and 2008. Patient characteristics and postoperative complications stratified by time periods are shown in Table 1 and Table 2, respectively.

**Table 1:** Patient characteristics (N = 4639).

Characteristic	2001–2002 n (%)	2003–2004 n (%)	2005–2006 n (%)	2007–2008 n (%)	P_Trend_
Overall	1574 (13)	1147 (9)	984 (8)	934 (7)	-
Age, Mean ± SD	64 ± 11	64 ± 10	64 ± 10	63 ± 10	0.23 ^†^
Male Sex	1094 (70)	812 (71)	712 (72)	695 (74)	0.0062 ^*^
White Race	1268 (81)	923 (80)	785 (80)	709 (76)	0.010 ^*^
BMI (mg/kg^2^), Mean ± SD	29 ± 5.6	30 ± 5.9	30 ± 5.8	30 ± 5.9	0.23 ^†^
Elective Surgery	706 (45)	741 (65)	466 (47)	382 (41)	0.0050
CAD Severity 1 Vessel 2 Vessel 3 Vessel	103 (7)	75 (7)	53 (5)	76 (8)	0.18 ^††^
	387 (25)	294 (26)	228 (23)	254 (27)	
	1084 (69)	778 (68)	703 (71)	604 (65)	
Left Main Disease	280 (18)	284 (25)	343 (35)	245 (26)	<0.0001 ^*^
Recent Smoker	424 (27)	356 (31)	301 (31)	322 (34)	0.0001 ^*^
Hypertension	1220 (78)	886 (77)	805 (82)	832 (89)	<0.0001 ^*^
Diabetes	592 (38)	436 (38)	368 (37)	405 (43)	0.017 ^*^
Heart Failure	251 (16)	312 (27)	296 (30)	236 (25)	<0.0001 ^*^
Dialysis	36 (2)	16 (1)	17 (2)	26 (3)	0.53 ^*^
PAD	199 (13)	173 (15)	179 (18)	166 (18)	<0.0001 ^*^
COPD	96 (6)	201 (18)	199 (20)	245 (26)	<0.0001 ^*^
Prior Stroke	107 (7)	99 (9)	84 (9)	93 (10)	0.0067 ^*^
Prior MI	691 (44)	541 (47)	455 (46)	470 (50)	0.0043 ^*^
Prior PCI	307 (19)	257 (22)	244 (25)	238 (25)	0.0001 ^*^

^*^ Cochran-Armitage Trend Test; ^†^ Linear Regression; ^††^ Chi-square Test for non-zero Correlation. BMI = body mass index; CAD = coronary artery disease; COPD = chronic obstructive pulmonary disease; MI = myocardial infarction; PAD = peripheral arterial disease; PCI = percutaneous coronary intervention; SD = standard deviation.

**Table 2:** Postoperative complications (N = 4639).

Complication	2001–2002 n (%)	2003–2004 n (%)	2005–2006 n (%)	2007–2008 n (%)	P_Trend _^*^
Myocardial Infarction	3 (<1)	3 (<1)	3 (<1)	3 (<1)	0.49
Stroke	23 (1)	27 (2)	11 (1)	11 (1)	0.30
ARDS	25 (2)	23 (2)	9 (1)	3 (<1)	0.0023
Pneumonia	27 (2)	31 (3)	26 (3)	17 (2)	0.65
Renal Failure	32 (2)	38 (3)	27 (3)	11 (1)	0.27
Operative Mortality	46 (3)	41 (4)	25 (3)	13 (1)	0.018

^*^ Cochran-Armitage Trend Test; ARDS = acute respiratory distress syndrome.

**Figure 1:**
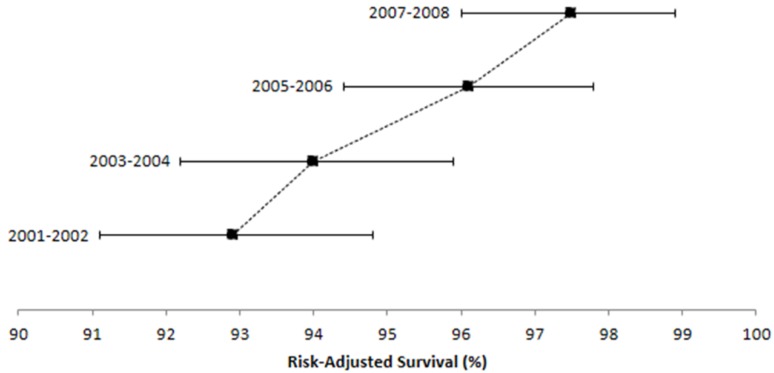
Efficiency-Outcome (EO) plot of risk-adjusted survival at six months ^*^.

^* ^95% CI noted by horizontal bars. Estimates shown were adjusted for age, sex, race, CAD severity, hypertension, heart failure, and prior stroke. CAD = coronary artery disease; CI = confidence interval.

**Figure 2:**
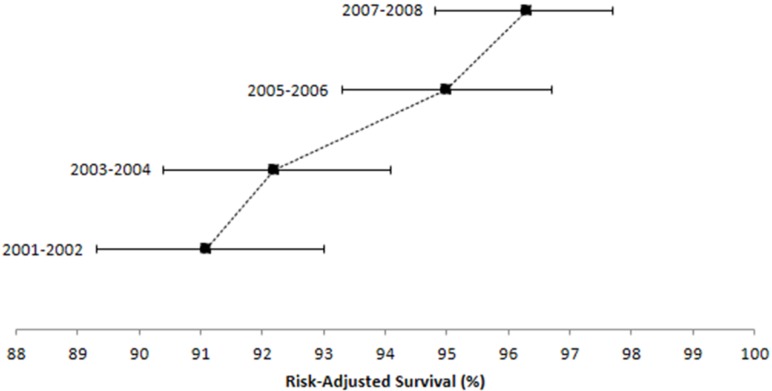
Efficiency-Outcome (EO) plot of risk-adjusted survival at one year ^*^.

^* ^95% CI noted by horizontal bars. Estimates shown were adjusted for age, sex, race, CAD severity, hypertension, heart failure, and prior stroke. CAD = coronary artery disease; CI = confidence interval.

Efficiency-outcome plots of 6-month and 1-year risk-adjusted survival are shown in Figure 1 and Figure 2, respectively. Six-month survival increased during the analysis period (2001–2002: adjusted survival = 92.9%, 95%CI = 91.1–94.8; 2007–2008: adjusted survival = 97.5%, 95%CI = 96.0–98.9). A similar trend was observed for 1-year survival estimates (2001–2002: adjusted survival = 91.1%, 95%CI = 88.9–93.3; 2007–2008: adjusted survival = 96.3%, 95%CI = 94.4–98.3).

Our model performed moderately well at differentiating patients who lived from those who died (Table 3). Furthermore, the overall percentage difference values for age, sex, race, CAD severity, hypertension, heart failure, and prior stroke were within the predefined equivalence region, indicating good model performance (Table 4). Although, statistically significant differences from the equivalence region were observed for sex and heart failure in the 2003–2004 time period, the maximum percentage differences for these variables were relatively small (sex: 3.9%; heart failure: 3.4%). Excluding these variables from the model did not substantively affect our adjusted survival estimates for this time period (6-month: 94.4%, 95%CI = 92.6–96.1; 1-year: 92.7%, 95%CI = 90.6–94.8).

**Table 3:** C-statistic and 95% confidence interval.

Year Period	Number of Usable Pairs	Number of Concordant Pairs	Number of Discordant Pairs	C-Statistic (95%CI)
2001–2002	82,566	593,780	258,786	0.70 (0.62–0.77)
2003–2004	324,328	214,520	109,798	0.66 (0.55–0.77)
2005–2006	141,552	105,692	35,860	0.75 (0.59–0.88)
2007–2008	88,635	60,128	28,507	0.68 (0.48–0.85)
2001–2010	4,934,832	3,659,189	1,275,643	0.74 (0.69–0.79)

CI = confidence interval.

**Table 4:** Differential cross-validation (model performance) for survival estimates.

Characteristic (Levels)	% Within Equivalence Region				
	2001–2002	2003–2004	2005–2006	2007–2008	2001–2008
Age (<65, ≥65)	100 ^*^	100 ^*^	100 ^*^	100 ^*^	100 ^*^
Sex (Male, Female)	100 ^*^	85 ^††^	100 ^*^	94 ^*^	100 ^*^
Race (Black, White)	100 ^*^	100 ^*^	100 ^*^	100 ^*^	100 ^*^
CAD Severity (1, ≥2 vessels)	100 ^*^	100 ^*^	100 ^*^	100 ^*^	100 ^*^
Hypertension (Yes, No)	100 ^*^	100 ^*^	100 ^*^	100 ^*^	100 ^*^
Heart Failure (Yes, No)	100 ^* ^	76 ^†† ^	100 ^*^	100 ^*^	100 ^*^
Prior Stroke (Yes, No)	100 ^*^	94 ^*^	100 ^*^	100 ^*^	100 ^*^

^* ^*p* > 0.05; ^† ^*p* ≤ 0.05; ^†† ^*p* < 0.01. CAD = coronary artery disease.

Statistically significant interactions by time were observed for sex (2003–2006) and heart failure (2003–2004) (Table 5). A sensitivity analysis removing these variables from the affected time periods did not substantially affect model estimates.

**Table 5:** Test for interaction effects by time.

Characteristic	2001–2002		2003–2004		2005–2006		2007–2008		2001–2008	
	χ^2^	*p*-value	χ^2^	*p*-value	χ^2^	*p*-value	χ^2^	*p*-value	χ^2^	*p*-value
Age	0.25	0.62	0.32	0.57	0.19	0.66	0.32	0.57	0.0007	0.98
Sex	1.05	0.31	7.18	0.0074	8.1	0.0044	2.8	0.095	6.93	0.0085
Race	3.0	0.084	0.85	0.36	1.4	0.24	0.0	0.99	2.23	0.14
2-vessel CAD	0.13	0.72	0.29	0.59	0.042	0.84	0.25	0.62	0.052	0.82
3-vessel CAD	0.28	0.60	0.17	0.68	0.69	0.41	0.043	0.84	0.53	0.47
Hypertension	0.038	0.85	1.2	0.28	0.012	0.91	3.1	0.079	0.035	0.85
Heart Failure	0.089	0.76	3.9	0.048	0.042	0.84	1.1	0.29	2.24	0.13
Prior Stroke	0.17	0.68	0.089	0.77	0.26	0.61	1.7	0.19	0.22	0.64
All Covariates	5.22	0.73	22	0.0046	12	0.16	9.1	0.33	15.84	0.045

χ^2^ = Chi-Square; CAD = coronary artery disease.

### 3.2. Discussion

Outcomes research is important for detecting performance changes and implementing quality improvements when necessary. Additionally, unwarranted, costly, and potentially harmful modifications to current clinical practice can be avoided. Focusing on patient-centered outcomes by incorporating individual risk factors and disease severity enables physicians to provide relevant information to patients and other stakeholders such as family members and caretakers. This information is particularly valuable for patients and their medical team who must decide on the best strategy for managing their postoperative care.

In this paper, we describe a simple technique to visualize risk-adjusted survival estimates that account for differences in case-mix severity. The application of this approach to regional centers allows for the localization of risk stratification rather than applying overly broad and non-specific models to their patient populations. Efficiency-outcome plots can be used to monitor performance over continuous time periods to visually gauge whether deviations in outcomes have occurred independent of case-mix. While confidence intervals are provided, this technique primarily serves as a graphical tool for monitoring quality over time. In the example provided from our institution, 6-month and 1-year risk-adjusted survival estimates improved for each consecutive 2-year time period, but especially between 2004 and 2005 (as indicated by the decreased slope). The observed improvements likely were attributable to better postoperative care (e.g., timely identification and management of complications), provider specialization, and hospital-wide quality control efforts.

Case-mix severity may change with time, potentially biasing the comparison of survival estimates between time periods. This was evident in our data as more patients in consecutive time periods presented with a larger percentage of comorbid conditions (e.g., heart failure, diabetes, hypertension, prior stroke, and prior myocardial infarction). Comparing performance solely on the basis of crude-survival figures may be misleading, especially when patients have been artificially selected to improve surgical outcomes. Additionally, efficiency-outcome plots are able to detect improvements or deteriorations after process changes in clinical care have been implemented. Although we have provided an example of how to monitor survival after CABG, this technique is equally applicable to other surgical procedures and medical interventions.

Prediction models for survival after CABG have recently been examined. A study using data from the New York State Cardiac Surgery Reporting System has developed a model for observed and predicted risk of death at years 1, 3, 5, and 7 after surgery [2]. However, data used in this analysis were specific to regions of New York and may have little interpretability to rural regions or other parts of the United States. Similarly, an analysis of data from the national STS Adult Cardiac Surgery Database has developed a risk prediction model for survival after CABG [1]. Notably, the applicability of this model to priority populations is limited as only 4% of the patients in this study were identified as black. The above studies highlight the shortcomings of applying generalized prediction models of survival to groups different from those in which they were originally constructed [3].

To date, several useful methods for plotting outcomes data (e.g., process charts, cumulative sum (CUSUM) charts, funnel plots, and resetting sequential probability ratio test (RSPRT) charts) to visually assess changes in risk over time for cardiovascular procedures have been presented in the literature [24,25,26]. Typically, these procedures have relied on parametric techniques such as likelihood-based scoring or the sequential probability ratio test. To the best of our knowledge, efficiency-outcome plots represent a unique application for generating continuous risk adjusted plots that account for censored data, independent of the parametric assumptions that underlie other commonly used graphical methods for risk assessment [24,25,26]. Efficiency-outcome plots also have the distinct advantage of tailoring the demographics to the center being assessed. The plots are simple to interpret and are easily generated using standard statistical software packages that compute multivariable product-limit survival probabilities. Furthermore, we have outlined how to cross-validate the model performance of efficiency-outcome plots using an equivalence region approach based on the percentage difference of fitted probabilities.

In the example provided, intra- and postoperative management of CABG have improved within our center. This likely represents a similar trend observed at other cardiovascular surgical facilities across the United States. However, there could be exceptions, especially within rural regions, where a focus on quality improvement is not emphasized. The advantage of efficiency-outcome plots is that they are able to identify, in a timely manner, departures from the norm. This will enable health care institutions to investigate the underlying cause of these changes and implement either reinforcing or corrective strategies. Furthermore, this method may be applied to other performance-related measures (e.g., patient satisfaction, return to work, quality of life, hospital acquired infections, operative mortality, operative time). We chose 6-month and 1-year survival for illustration purposes only.

Specifically, the focus of the current manuscript was on introducing this novel method and providing our institution as an example. Given that the technique adjusts for case mix, the resulting plots may be compared between institutions as well. While, the latter is beyond the scope of the current study, it would be interesting to use efficiency-outcome plots to compare performance across institutions in a future study.

### 3.3. Limitations

The methodology described in our analysis has several limitations inherent to modeling censored survival data and must be considered when applying this technique. The estimation of adjusted survival probabilities assumes that the relationship between the log cumulative hazard and a set of covariates must be approximately linear [15]. Furthermore, censoring must be non-informative (*i.e*., not related to the probability of an event occurring). The latter assumption poses a limitation for any time-to-event model and must be addressed in the design phase. In some cases, adding interaction terms (including time) to our model may be necessary when the underlying data seriously violate the parallel hazard assumption [23]. Additionally, redundant and highly correlative variables may lead to unstable estimates and should be avoided.

Contextual factors including surgeon training, experience, caseload, and turnover may have explained differences in quality measures over time. However, adjusting for these factors in our model would have led to over-adjustment and possibly masked any underlying differences in institutional practice. This would have been counterintuitive to the purpose of our model, which is to monitor changes in healthcare performance independent of changes in patient characteristics (e.g., case-mix).

Efficiency-outcome plots rely on semi-parametric survival analysis methodology and do not have a simple, closed mathematical form. Consequently, estimates are derived using an iterative computer algorithm. However, such algorithms are commonly available in most standard statistical packages.

## 4. Conclusions

In this paper, we describe a practical technique to graphically monitor efficiency of care and consequent risk over time, and provide an example of how this model can be used in the cardiovascular surgery setting to improve overall healthcare performance. Efficiency-outcome plots allow individual centers to continuously assess quality measures and determine when process changes result in improved or poorer outcomes. Future research may benefit from active surveillance of morbidity and mortality following CABG to provide real-time quality measurements.
